# Sarcopenic Dysphagia, Malnutrition, and Oral Frailty in Elderly: A Comprehensive Review

**DOI:** 10.3390/nu14050982

**Published:** 2022-02-25

**Authors:** Alessandro de Sire, Martina Ferrillo, Lorenzo Lippi, Francesco Agostini, Roberto de Sire, Paola Emilia Ferrara, Giuseppe Raguso, Sergio Riso, Andrea Roccuzzo, Gianpaolo Ronconi, Marco Invernizzi, Mario Migliario

**Affiliations:** 1 Department of Medical and Surgical Sciences, University of Catanzaro “Magna Graecia”, 88100 Catanzaro, Italy; 2 Department of Health Sciences, University of Catanzaro “Magna Graecia”, 88100 Catanzaro, Italy; 3 Department of Health Sciences, University of Eastern Piedmont “A. Avogadro”, 28100 Novara, Italy; lorenzolippi.mt@gmail.com (L.L.); marco.invernizzi@med.uniupo.it (M.I.); 4 Department of Anatomical and Histological Sciences, Legal Medicine and Orthopedics, Sapienza University, 00185 Rome, Italy; francescoagostini.ff@gmail.com; 5 Department of Clinical Medicine and Surgery, University Federico II of Naples, 80126 Naples, Italy; roberto.desire@libero.it; 6 University Polyclinic Foundation Agostino Gemelli IRCSS, Catholic University of Sacred Heart, 00168 Rome, Italy; paolaemilia.ferrara@policlinicogemelli.it (P.E.F.); gianpaolo.ronconi@policlinicogemelli.it (G.R.); 7 Department of Otolaryngology-Head and Neck Surgery, University of Verona, 37129 Verona, Italy; ragusogiu@gmail.com; 8 Dietetic and Clinical Nutrition Unit, Maggiore della Carità Hospital, 28100 Novara, Italy; sergio.riso@maggioreosp.novara.it; 9 Department of Periodontology, School of Dental Medicine, University of Bern, Freiburgstrasse 7, 3010 Bern, Switzerland; andrea.roccuzzo@zmk.unibe.ch; 10 Department of Oral and Maxillofacial Surgery, Copenhagen University Hospital (Rigshospitalet), 2100 Copenhagen, Denmark; 11 Translational Medicine, Dipartimento Attività Integrate Ricerca e Innovazione (DAIRI), Azienda Ospedaliera SS. Antonio e Biagio e Cesare Arrigo, 15121 Alessandria, Italy; 12 Dental Clinic, Department of Translational Medicine, University of Eastern Piedmont, 28100 Novara, Italy; mario.migliario@med.uniupo.it

**Keywords:** sarcopenic dysphagia, sarcopenia, dysphagia, malnutrition, oral health, osteoporosis, elderly

## Abstract

Frailty is a highly prevalent condition in the elderly that has been increasingly considered as a crucial public health issue, due to the strict correlation with a higher risk of fragility fractures, hospitalization, and mortality. Among the age-related diseases, sarcopenia and dysphagia are two common pathological conditions in frail older people and could coexist leading to dehydration and malnutrition in these subjects. “Sarcopenic dysphagia” is a complex condition characterized by deglutition impairment due to the loss of mass and strength of swallowing muscles and might be also related to poor oral health status. Moreover, the aging process is strictly related to poor oral health status due to direct impairment of the immune system and wound healing and physical and cognitive impairment might indirectly influence older people’s ability to carry out adequate oral hygiene. Therefore, poor oral health might affect nutrient intake, leading to malnutrition and, consequently, to frailty. In this scenario, sarcopenia, dysphagia, and oral health are closely linked sharing common pathophysiological pathways, disabling sequelae, and frailty. Thus, the aim of the present comprehensive review is to describe the correlation among sarcopenic dysphagia, malnutrition, and oral frailty, characterizing their phenotypically overlapping features, to propose a comprehensive and effective management of elderly frail subjects.

## 1. Introduction

In the last decades, worldwide life expectancy has increased and the proportion of older adults relative to other age groups has continued to grow resulting in ageing of the society, especially in the developed countries [1,2,3]. Life courses of health and functional status in older people are related to their genetics and environmental backgrounds, as well as other physical and psychological factors [4]. Indeed, a common feature in elderly subjects is the progressive decline in several physiological functions, which might lead to an increased risk of sarcopenia, dysphagia, osteoporosis, and frailty [5,6,7,8].

In this context, frailty is a complex and multifaceted public health issue highly prevalent in older adults leading to increased direct and direct sanitary costs and is strictly correlated with a higher risk of falling, fragility fractures and consequent disability, hospitalization, and mortality [5,9,10,11]. A consistent percentage of frail subjects might present with sarcopenia, a clinical condition, characterized by a reduction in muscle mass, muscle strength, and physical performance [12]. Sarcopenia typically occurs during the fifth decade of life affecting from 9.9 to 40.4% of older people [13] and inducing a muscle mass and strength decline, with a consequent need of a prompt diagnosis and a rehabilitative intervention [6,14,15]. Moreover, it has been recently shown that sarcopenia could be considered as an independent risk factor for dysphagia, reducing the strength of swallowing muscles [5,16,17]. Dysphagia is a dysfunction of the digestive system, characterized by an impairment in chewing and swallowing, with absence or prolonged transit of food or liquids in the upper digestive tract [18]. The oropharyngeal swallowing process involves a coordinated set of neuromuscular actions allowing the transit of the bolus from the oral cavity to the upper esophageal sphincter and is commonly described in three different phases: oral, pharyngeal, and esophageal [19]. Hence, oropharyngeal dysphagia could result in an ineffective deglutition, leading to dehydration and malnutrition with a consequent increased risk of muscle mass loss [20]. In this scenario, sarcopenia and dysphagia share several risk factors and their pathological coexistence has captured the interest of the scientific community in the last few years. Moreover, both conditions could be considered both risk and predictive factors at the same time [17]. Thus, the concept of “sarcopenic dysphagia”, a pathological condition caused by a loss of mass and strength of swallowing-related muscles, has been recently proposed and, in light of its detrimental effects on health-related quality of life (HRQoL) and disability requires a multidisciplinary approach and effective management in elderly subjects [21,22,23].

As previously described, dysphagia might lead to malnutrition through several pathological mechanisms [24,25] with consequent increased risk of developing sarcopenia [26]. At the same time, malnutrition can be caused by a variety of factors including difficulty eating, reduced mobility, psychological stress, and poor access to healthcare, oral health care, and social services [27]. Individuals with impaired masticatory ability usually avoid foods that are difficult to chew, including raw vegetables or fruits. Indeed, an insufficient intake of fibers, linoleic acid, potassium, calcium, magnesium, zinc, selenium, vitamins D, E and K, folate, biotin, and molybdenum have been observed in older adults with dysphagia with reported chewing and swallowing impairments [24,28]. Among all these pathophysiological mechanisms underpinning malnutrition, poor oral hygiene could play a key role in the development of this condition, increasing the risk of progressive periodontal disease and dental decay, as shown in other chronic conditions [29,30,31,32]. Indeed, oropharyngeal functional decline culminates in a loss of independence in self-care abilities, including less attention for oral care [33] and activities of daily living (ADL), such as brushing teeth or dentures or periodic visits to the dentist [34]. Moreover, oral cancer, dry mouth (xerostomia), and pathological denture-related conditions (including oral candidiasis and denture stomatitis) [3,35] are more frequent in the elderly [36], resulting in variable degrees of oral disability. Lastly, a reduction in appetite might occur because of decrease in smell and taste senses, leading to a loss of pleasure in eating, which is another well-known risk factor for malnutrition [37,38]. In conclusion, poor oral health might affect nutrient intake, leading to malnutrition and, consequently, to frailty.

**Figure 1:**
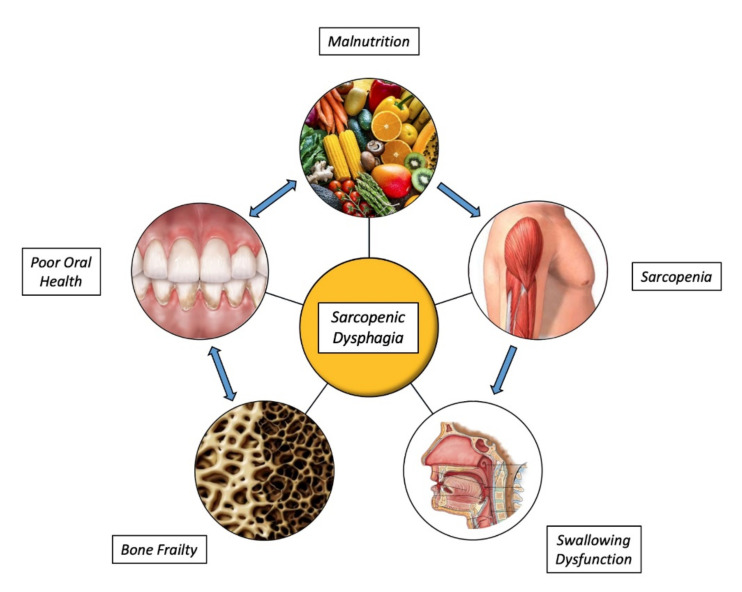
Overview of the vicious circle among malnutrition, sarcopenia, swallowing dysfunction, bone frailty, and poor oral health in older subjects.

In this scenario, age-related conditions are a growing issue and need comprehensive and multidisciplinary interventions for a prompt and effective management. sarcopenia, dysphagia, sarcopenic dysphagia, and oral health seem to be closely linked and share common pathophysiological pathways, overlapping features and disabling sequelae all leading to frailty (see Figure 1).

However, at present, few reports in the literature focused on these specific issues, their impact on disability and the pathophysiological synergies of these different conditions of older adults.

Therefore, by this comprehensive review we sought to describe the state-of-art about the overlapping features of sarcopenia, sarcopenic dysphagia, malnutrition, and oral frailty in the elderly and their correlation to define the correct framework for an optimal management of these complex pathological conditions.

## 2. Malnutrition and Aging: A Close but Unclear Link in the Elderly

An adequate nutritional status and physical activity are cornerstones to preserve functioning, wellbeing, and HRQoL in older people, according to the Healthy Aging policy framework of the World Health Organization [39]. However, nutritional disorders are a critical burden in the elderly, affecting physical function and global health with detrimental consequences in well-being and sanitary costs [40].

According to the European Society for Clinical Nutrition and Metabolism (ESPEN), diagnostic criteria for malnutrition are defined by a body mass index (BMI) of <18.5 kg/m^2^ or by meeting two of these three criteria: unintentional weight loss of more than 10% (or more than 5% over the last three months), BMI lower 22 kg/m^2^ (or lower than 20 kg/m^2^ in persons over 70 years old), or a low fat-free mass index (FFMI) score (FFMI < 15 in women and FFMI < 17 kg/m^2^ in men) [41].

In recent years, malnutrition prevalence is increasing worldwide due to the aging of the population and the increasing prevalence of age-related pathological conditions [42]. To date, a recent meta-analysis including over 110,000 older persons underlined that malnutrition rate might range between 6% (95% CI, 4.6–7.5) and 29.4% (95% CI, 21.7–36.9) based on the health care setting [43]. In more detail, rehabilitation and subacute care settings were associated with a higher prevalence of malnutrition [43]. In accordance, Wojzischke et al. [44] reported similar results, while approximately 47% (40–54) of geriatric rehabilitation patients were at risk of malnutrition.

Albeit several pathophysiological mechanisms underpinning the strict association between malnutrition and aging have been hypothesized [45], the gap of knowledge remains still consistent. Physical function impairment, social and environmental conditions, acute and chronic diseases, and pharmacological treatments have been identified as independent risk factors potentially responsible to generate malnutrition in the elderly [45]. Moreover, as previously suggested, malnutrition in the elderly might be related to dental problems or to dysphagia due to a reduced performance in swallowing functions that might affect oral intake [46,47]. Therefore, patients who could not achieve full oral intake with support or supplemental strategies might undergo a catabolic state with detrimental consequences in several body tissues tropism and systemic inflammation [48].

The relationship between oral health and nutrition is multidirectional: on the one hand, oral health problems (e.g., tooth loss, toothache) could be contributing factors to malnutrition, through the reduction of chewing skills (e.g., edentulism, dry mouth) [49,50]. In this context, Joshipura et al. showed that edentulous people consumed fewer vegetables, less fiber and carotene and more cholesterol, saturated fat, and calories than did participants with 25 or more teeth [51]; on the other hand, poor dietary intake increased the risk of periodontal disease. Indeed, inverse associations were found between fatty acids, vitamin C, vitamin E, beta-carotene, fiber, calcium, dairy, fruits, and vegetables and risk of periodontal disease [52,53]. Moreover, a strict relationship between the development of periodontal disease and the diet-borne systemic inflammation was supposed [53]. Indeed, nutrients consumption of dairy, fruits and vegetables, fiber, calcium, antioxidants, and fatty acids might regulate the immune-mediated inflammatory responses, starting and propagating pro-inflammatory mechanisms, which are basis of periodontal disease development [54].

Therefore, an early identification of malnutrition is mandatory to optimize the complex management of elderly patients and to prevent the negative clinical consequences of malnutrition. Several screening and grading tools have been proposed to better characterize malnutrition, including Mini Nutritional Assessment (MNA) [55], Malnutrition Screening Tool [56,57], Seniors in the Community: Risk Evaluation for Eating and Nutrition (SCREEN II) [58], Malnutrition Universal Screening Tool [32,59], Simplified Nutrition Assessment Questionnaire (SNAQ) [60], and Nutritional Risk Screening (NRS) [61]. Despite nutritional screening should be routinely performed in the hospital setting, there is still a lack of consensus on the optimal tool to be used in clinical practice to promote the early identification of this condition and an effective and tailored treatment.

To date, it has been widely recognized that malnutrition is related to poor health outcomes due to its clinical consequences in both acute and chronic diseases [62,63]. Moreover, malnutrition is currently considered as one of the main modifiable prognostic factors for worsening outcomes and mortality in elderly patients [64,65]. Lastly, a nutritional deficiency of micronutrients (vitamin D [66,67], vitamin C [66], vitamin B12 and folate [67,68], vitamins A and E [69], vitamin B6 [70], selenium, zinc, magnesium [71], and copper [72]) has been reported to have a potential role in the immune regulation in the elderly. In this scenario, malnutrition is considered as a risk factor for osteoporosis, sarcopenia, and frailty [73,74]. To date, several studies [74,75,76] highlighted a correlation between protein intake and calcium-phosphate or bone metabolism reporting that deficient protein supply might affect calcium homeostasis. Moreover, protein deficiency might downregulate insulin-like growth factor-I (IGF-I) production which plays a key anabolic role in skeletal muscle, cartilage, and bone [77].

Concurrently, malnutrition consequences might reflect on musculoskeletal system with crucial implications on functional performances and HRQoL of frail elderly patients [78]. Thus, the role of nutritional interventions in the elderly has been widely investigated with increasing evidence supporting the positive role of oral supplementation to optimize the rehabilitative path of frail patients [79].

However, it should be noted that tailored treatment should be proposed in patients affected by concurrent conditions. In particular, it should be noted that dysphagia, malnutrition, and oral frailty frequently coexistent in the elderly [80]. Therefore, nutritional intervention should take into account individual swallowing capacities [81]. Moreover, due to the detrimental effects of malnutrition in musculoskeletal system, sarcopenia and frailty syndrome should be screened in patients with malnutrition due to the high prevalence of these concurrent age-related conditions and the needing for enhancing synergism among therapeutic interventions [82].

Taken together, the evidence reported put in light the needing for early screening for malnutrition in the elderly to minimize malnutrition complications and set-up multi-disciplinary strategies to treat this complex and disabling condition and prevent its health-related consequences.

## 3. Dysphagia in Older Subjects

Swallowing problems have been considered as a growing health concern for the elderly, being a major cause of malnutrition, dehydration, aspiration pneumonia or even death due to asphyxiation [83]. Prevalence ranges from 16% in people aged 70–79 years to 33% in subjects aged more than 80 years [20], reaching 60% in geriatric populations residing in community dwelling settings and nursing homes [84].

Dysphagia is defined as a difficulty in eating and swallowing, characterized by impaired or prolonged transit of food or liquids from the oral cavity to the esophagus [18]. The swallowing process could be divided into four distinct phases: oral preparatory, oral transport, pharyngeal, and esophageal phase. An impairment in any of these phases may lead to dysphagia [85]. The swallowing function may be altered, in every single phase, by the age-related reduction of tissue elasticity, cervical spine changes, oropharyngeal disorders, decrease of oral moisture, and sensory impairments, such as reduction of smell and taste [19]. The coexistence of sarcopenia and dysphagia has recently attracted a considerable amount of interest in the scientific literature, considering that older people with dysphagia might present a loss of muscle mass and strength in both generalized skeletal muscles and swallowing-related muscles [22]. Indeed, this age-related loss of muscle mass might be manifested as a decrease in the thickness of tongue, geniohyoid muscle [86], pharyngeal wall [87], and a reduction of tongue pressure [88] and weaker pharyngeal contractility [89].

Dysphagia management requires a multidisciplinary approach focusing at first on early diagnosis to prevent potential complications [90]. Hence, dysphagia screening can be performed using validated questionnaires designed to rapidly detect signs and symptoms of swallowing impairment, such as the 10-Item Eating Assessment Tool (EAT-10) [91,92]. This tool is composed of 10 items, each one describing a specific risk condition to be scored from 0 (absence of problem) to 4 (severe problem), and a total score of 3 or higher suggests an abnormal swallowing function. This screening tool reported a high specificity (96.8% if ≥3 and 98.4% if ≥4) for detecting dysphagia in the elderly as shown in a study performed on 534 older people referred to a Rehabilitation Unit after total hip or knee arthroplasty [5]. Another screening test commonly used in the clinical practice is the modified water swallowing test (MWST), which shows a sensitivity and a specificity of 70% and 88%, respectively, for detecting aspiration [93,94]. Similarly, a wide variability of screening tools showed excellent sensitivity and specificity in the assessment of patients with dysphagia, including volume–viscosity swallow test, pharyngo-esophageal manometry, voluntary cough airflow, maximum tongue pressure, surface electromyography, real-time magnetic resonance imaging [95]. These tests might be associated with each other in order to improve specificity and sensibility for a more precise dysphagia assessment [93,94,95].

Moreover, several rating scales are used to assess the oral intake level of patients, which correlates with deglutition ability, such as the Functional Oral Intake Scale (FOIS), Food Intake Level Scale (FILS), neuromuscular disease swallowing status scale, and Sydney Swallow Questionnaire [96,97].

However, despite these tools being the first-line use in common clinical practice, the instrumental evaluation is mandatory to confirm the diagnosis of dysphagia [20]. The video-fluoroscopic swallowing study (VFSS), also known as modified barium study, is the only diagnostic tool that assesses all four phases of swallowing. It could detect oral and pharyngeal motility problems, ascertain presence of aspiration or penetration, assess the swallow speed and evaluate postural changes and their effect on aspiration/penetration [20]. A video-fluoroscopic study performed on 731 patients complaining of swallowing symptoms showed prolonged oral transit time and aspiration after swallowing in elderly dysphagic patients. Similarly, a study performed on 132 patients with swallowing difficulties using fluoroscopic imaging showed that male sarcopenic patients had lower laryngeal upward movements during swallowing and wider pharyngeal areas compared to healthy controls [88].

Another instrumental technique for dysphagia assessment is the flexible endoscopic evaluation of swallowing (FEES) that allows a direct visualization of the laryngopharynx, whereas patients are asked to eat different consistencies of food with food coloring [98]. It is a very useful tool to assess the presence of penetration or aspiration residue in the valleculate and pyriform sinuses, despite it is limited in exploring the oral and esophageal phases of deglutition. Giraldo-Cadavid et al. showed that aspiration detected by FEES and an age > 65 years were two independent predictors of mortality in 148 patients with oropharyngeal dysphagia [98].

In conclusion, dysphagia is a common and disabling issue in the elderly and need to be managed through a complex multidisciplinary approach, starting from early diagnosis and involving several health professionals such as geriatric, otorhinolaryngoiatric, physical and rehabilitation physicians, nutritionists, dentists, and speech-language pathologists in order to plan the most effective treatment.

## 4. Oral Frailty: A Detrimental Issue

The oral cavity is the first part of the digestive tract and is involved in several functions including biting the food, chewing, adding saliva for bolus formation and transporting it into the stomach [99]. Poor oral health seems to be strictly related to aging and could be considered as an indicator of frailty [100,101]. It has been shown that poor oral health is associated with poor diet quantity and quality in older adults [102], leading to a consequent reduction of fruits, vegetables, and fibers intake leading to an increased risk of malnutrition [103]. Moreover, the number of teeth is significantly associated with the number of food items that older persons able to eat [103]. Indeed, tooth loss could influence the selection of food of reduced consistency and consequent loss of pleasure in eating [25], explaining the relationship between tooth loss and poor nutritional status in the elderly [104].

In this scenario, Hussein et al. [105] in 2021 performed a systematic review with meta-analysis showing that edentulous patients had a 9.5% higher risk of malnutrition than healthy subjects, evidencing a lack of specific nutrients, that could lead to several disorders. The authors reported that older adults with chewing impairment had twice the risk of malnutrition and those with no daily teeth or denture cleaning had a 52.6% higher risk of malnutrition compared to control subjects. Iwasaki and colleagues [106] investigated the nutritional status among 1054 community-dwelling older adults, reporting that oral frailty is defined as the number of remaining teeth, masticatory performance, articulatory oral motor skill, low tongue pressure and eating and swallowing impairment was present in 20.4% of the patients assessed. Study participants with oral frailty showed a higher odd of more severe malnutrition, evaluated using the Mini-Nutritional Assessment—Short Form and serum levels of albumin. Furthermore, it should be highlighted that a poor oral health status (in particular edentulism) increased the difficulty in eating hard foods, with a consumption of mashed food and decreasing eating pleasure, leading to a higher risk of malnutrition [107].

Another age-related condition that could affect oral health in the elderly is oral dryness, with a negative impact on oral health status and HRQoL [108,109,110]. Xerostomia is considered as the subjective sensation of oral dryness, ranging from 17% to 40% among community-dwelling elderly and from 20% to 72% in institutionalized older people [111]. The prevalence of dry mouth increases with increasing numbers of medications used [109,112], especially if used in combination [113]. More than 400 medications could cause xerostomia, including antidepressants, proton pump inhibitors, antihypertensives, antipsychotics, diuretics, and antineoplastics. Xerostomia could also be caused by autoimmune conditions, as the Sjögren’s Syndrome [114], radiation therapy for cancers of the head and neck [115], dehydration [116] and infection as hepatitis C virus (HCV) [117]. Moreover, saliva plays a key role in neutralizing potentially damaging food acids and enhancing the ability to taste food and speech facilitation [118]. It contains several enzymes, which start the digestion process, and antibacterial, antifungal, and antiviral agents, which are extremely helpful to prevent oral infections [119]. Older people often present with a reduced salivary flow, with negative consequences for oral health, including dysgeusia, halitosis, burning mouth, oral pain, difficulty in chewing and swallowing, speech impairment, and an increased risk of fungal infections, demineralization/caries, and periodontitis [120,121,122,123,124].

Periodontal disease is a chronic inflammatory pathological condition affecting the tooth-supporting soft and hard tissues, which left untreated leads to tooth mobility and tooth loss [56,125]. The constant deposit of bacterial biofilm on the teeth triggers a chronic inflammatory condition ranging from a reversible low-level (gingivitis) to irreversible higher level of inflammation (periodontitis) and tooth mobility/loss [126]. In this context, microbial products and inflammatory mediators might enter the systemic circulation and reach distant organs, supporting the genesis of systemic pathologies [127,128,129,130]. Nutrition is a critical determinant of immune responses [131] because nutrients derived from food sources show a strong interaction with the immune system cells [132] and nutritional deficiency might impair the immune response and predispose the individual to infection [133,134]. In more detail, a low intake of vitamin A, E, C, B6, and B12, pantothenic acid, riboflavin, and folate act on DNA and RNA synthesis, cellular metabolism, and antioxidant activity and a low intake of these micronutrients might affect the host defenses [135,136]. The result is a state of chronic inflammation that might induce an intrinsic production of glucocorticoids and proinflammatory cytokines with consequent body weight loss and skeletal muscle depletion [137,138] Moreover, the high levels of pro-inflammatory cytokines (i.e., interleukin-6 and TNF-α) have been associated with reduced muscle mass and muscle strength [139,140]. Hence, poor oral health conditions characterized by high values of plaque and bleeding on probing scores might be strictly related to dysphagia, sarcopenia, and malnutrition, sharing some pathophysiological mechanisms and phenotypic manifestations with these pathological conditions.

Oral health status is also considered to be a factor associated with sarcopenia [141] and dysphagia [142], and improvement of the oral status and function might be important to conduct dysphagia rehabilitation. Poor oral health status may induce difficulties in chewing and swallowing in older people, as well as malnutrition and consequent sarcopenia. Authors showed a strong relationship between dysphagia and low salivary flow [143], which could induce both a dry feeling in the mouth and a defect in lubrication and the cohesion of the bolus [143,144]. Dysphagia associated with salivary hypofunction can cause a loss of appetite and a restricted choice of dietary intake [143].

Moreover, much attention has been focused on the relationship between oral health and sarcopenia and it was assumed that impaired oral health leads to sarcopenia. In this context, some reports have shown relationships between oral health and handgrip strength, walking speed, and skeletal muscle mass which are measurements used in the diagnosis of sarcopenia [145,146,147].

Moreover, given the recent evidence underlining a strict muscle–skeletal crosstalk, intriguing implications have been suggested in the relationship between oral health and bone frailty [148,149].

Therefore, in conclusion, impaired oral health can lead to malnutrition and sarcopenia, which can, in turn, cause dysphagia, resulting in a negative cycle that worsens the patient’s general condition.

## 5. Sarcopenic Dysphagia: An Old and New Concept

The term “sarcopenic dysphagia” has been used for the first time in 2012 by Kuroda and Kuroda [21] to define a swallowing impairment due to both systemic and swallowing muscles sarcopenia [22]. To date, this topic has been rising a growing interest in the scientific field with four academic societies that recently published a position paper to better characterize the definition and diagnosis of sarcopenic dysphagia [150]. In this scenario, a progressive decline in skeletal muscle mass in the elderly is widely documented in literature [151,152,153,154,155]. However, this phenomenon might be extremely burdensome in frail patients and might affect even swallowing muscle, including the tongue, geniohyoid muscle, and pharyngeal muscles with negative consequences in terms of swallowing function and consequent increased risk of dysphagia [87,156,157,158].

Therefore, sarcopenic dysphagia is characterized by specific differences from presbyphagia in elderly. In more detail, although presbyphagia is associated with age-related decline of swallowing mechanisms, sarcopenic dysphagia might be related to a further decline in swallowing muscle strength due to an impairment of whole-body skeletal muscle strength associated with a reduction in swallowing function [22]. The negative bond of events characterizing the evolution from presbyphagia to sarcopenic dysphagia has been not fully understood; however, it has been proposed that energy intake reduction and acute diseases might severely affect the risk for sarcopenic dysphagia in elderly [17,159].

In this context, Ogawa et al. [160] identified the cross-sectional area of the tongue muscle as the most specific factor to assess sarcopenic dysphagia. Moreover, togue muscle area of brightness assessed with ultrasound technique seem to be an independent risk factor for sarcopenic dysphagia [160]. On the other hand, Maeda et al. [17] reported that skeletal muscle index, Barthel Index and BMI were significantly related to sarcopenic dysphagia, supporting the growing evidence on the similar pathophysiological mechanisms underpinning these conditions, with a detrimental synergism in terms of frailty in older people [161,162]. Furthermore, the strict association between sarcopenia and dysphagia has been underlined by the recent systematic review by Zhao et al. [163], that reported a significant association between sarcopenia and dysphagia independently by the diagnostic criteria [163].

However, sarcopenic dysphagia is not simply identified by a concomitant diagnosis of sarcopenia and dysphagia, but it has been characterized by specific diagnostic criteria recently assessed by Mori et al. [164]. The authors emphasized the need for a precise tool to identify sarcopenic dysphagia, aiming at developing specific strategies to counteract this progressive and disabling condition. The authors proposed a diagnostic algorithm consisting of five different items including dysphagia diagnosis, sarcopenia diagnosis, imaging test consistent with loss of swallowing-muscles mass, no other possible cause of dysphagia, or if any, not considered the main cause [164]. Based on these criteria, the authors identified three potential diagnostic categories: probable sarcopenia dysphagia, possible sarcopenic dysphagia, and no sarcopenic dysphagia [164].

However, it has been demonstrated that in stroke patients older than 65 years old, the swallowing muscle mass might be decreased even without a direct neurological deficit, mainly due to the high risk for malnutrition and oral intake reduction [48,165]. In particular, it has been reported that despite stroke-related dysphagia might be associated with common neurological symptoms including dysarthria, aphasia, apraxia, and facial palsy, concurrent or overlapping sarcopenic dysphagia might be related to pre-injury comorbidity, acute illness, immobilization, and inadequate energy intake that might affect post-stroke patients with a major latency of onset but better reversibility with a specific therapeutic intervention [165].

On the other hand, sarcopenic dysphagia has been also reported after severe COVID-19 infection in non-intubated elderly patients [166], leading to an intriguing interest on the screening strategies and therapeutic rehabilitation approach in COVID-19 survivors [167,168].

To date, several barriers have been identified in routine clinical screening of sarcopenia, including the lack of confidence in health care workers in the screening tools, the underestimation of the disease, the lack of specific services to manage sarcopenic patients, or limitations to access these specific services [169,170,171]. As a result, sarcopenic dysphagia screening has not been fully introduced in routine clinical practice, albeit recent studies [172,173,174,175] have emphasized the need for a specific therapeutic intervention including rehabilitation in this condition.

Taken together, these findings underlined that sarcopenic dysphagia is a common and disabling condition in elderly patients. Specific diagnostic criteria have been proposed for the early identification of this condition, and ultrasound imaging might be a useful tool to assess swallowing muscle mass and muscle quality [160].

In this scenario, clinicians should consider the strict associations among sarcopenia, dysphagia, and malnutrition in the therapeutic path of sarcopenic dysphagic patients in order to optimize the comprehensive management of elderly patients.

Moreover, precise identification of these concurrent conditions might have a key role in the optimal treatment strategy prescription directly targeting functional impairment related to these conditions and etiological causes at the basis of the onset of the disease.

**Figure 2:**
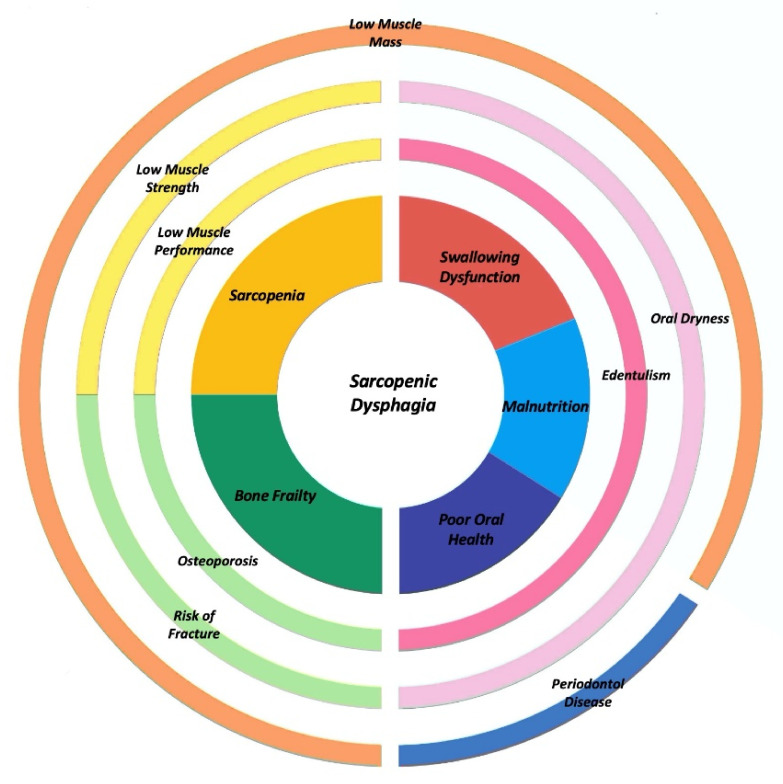
Sarcopenic dysphagia: overlapping features among different age-related conditions.

Figure 2 describes the overlapping features among different age-related conditions, underlining the key role of sarcopenic dysphagia.

Lastly, a routine clinical screening to assess sarcopenic dysphagia should be introduced in the clinical practice to support an early rehabilitative treatment and improve functional outcomes in sarcopenic elderly patients, potentially preventing malnutrition and frailty along with their burdensome consequences.

## 6. Nutritional Supplementation to Counteract Sarcopenic Dysphagia

Nutritional interventions are a cornerstone of the integrated therapeutic path aimed at counteracting sarcopenia and malnutrition in the elderly [176,177]. In more detail, the ESPEN [176] recently recommended an energy intake of 30 kcal/kg of body weight/day and a protein intake of at least 1.0 g/kg body weight/day in the elderly. However, the protein intake may reach 1.2–1.5 g/kg body weight/day in acute or chronic illness [176]. In sarcopenic patients, it has been shown that 1.2 g/ideal body weight/day (kg) protein intake might be effective in improving tongue muscle strength [178]. Along with the daily protein intake, also the daily caloric intake should be strictly monitored in elderly patients given the high risk of malnutrition in these subjects, especially in dysphagic ones [26,179]. In more detail, it has been suggested that a caloric intake ≥ 35 kcal/IBW/day (kg) is needed to adequately treat patients with sarcopenic dysphagia [150]. Furthermore, it should be noted that the reaching of an optimal energy and protein intake could be extremely challenging in these patients not only for the well-known swallowing impairment but also for the high prevalence of concomitant age-related disabling conditions [174]. Therefore, a specific therapeutic approach should be tailored to the patient’s needs and enteral or parenteral nutrition might be considered especially in post-acute patients [103]. However, conflicting data were reported for both enteral and parenteral nutrition solutions and oral feeding should be preferred if possible [180,181]. In this scenario, food texture modifications might be used to reduce the risk of inhalation and should be adapted to the swallowing deficiencies [182]. Moreover, an adequate nutritional supplementation might provide significant improvements in both macronutrients and micronutrients intake [183,184]. Indeed, promising results were reported in terms of vitamin D supplementation, which might have a crucial role not only on musculoskeletal health but also on immune system regulation with intriguing implications in several pathological conditions of the elderly [185,186].

To date, few studies assessed the effect of nutritional supplementation in patients with sarcopenic dysphagia reporting promising results [187,188,189,190]. However, it should be noted that nutritional supplements should be considered in an integrated multidisciplinary treatment including an adequate physical exercise [191,192,193]. In recent years, rehabilitation nutrition, a recent integrated approach aimed to counteract the effects of aging on the skeletal muscle system, has been proposed to optimize functional outcomes in the elderly [194]. It is based on a precise assessment of nutritional disorders, sarcopenia and potential deficits in nutritional intake [159]. More in detail, the close synergism between nutrition and rehabilitations has been deeply investigated in the past few decades [82,195]. Moreover, several papers supported the strong correlation between nutritional status and functional outcomes, and the greater improvement in these outcomes in sarcopenic patients obtained with combined nutritional and exercise interventions compared instead of single ones [196].

Despite these findings, good-quality clinical trials assessing the role of specific nutritional supplementation in sarcopenic dysphagic patients are still lacking. Moreover, there is still a gap of knowledge concerning the effect of physical exercise and nutritional supplementation in patients with sarcopenic dysphagia. Therefore, further research targeting these specific patients is still warranted to establish to guide clinicians in the management of frail subjects at risk of sarcopenic dysphagia.

## 7. Oropharyngeal Rehabilitation

Dysphagia and malnutrition are two major issues in the elderly with a negative impact on several pathological conditions, including cardiovascular disorders, cognitive status impairment, immune system downregulation, pressure ulcers, and skeletal muscle system worsening [197,198]. Therefore, effective interventions aimed at improving the nutritional intake and counter malnutrition are mandatory not only to prevent dysphagia-related complications (such as aspiration pneumonia) but also to improve the general health status and the HRQoL of older patients [199].

In this context, oropharyngeal rehabilitation is a relatively new concept in the complex multidisciplinary approach of dysphagia and swallowing disorders. This rehabilitative approach is characterized by several interventions including functional training, compensatory maneuvers, postural adjustments, swallowing maneuvers and diet modifications [156,200,201]. In more detail, postural adaptations might have a crucial role in airways closure and in reducing the risk of inhalations in order to improve the speed and safety of swallowing [201]. Thus, the posture of sitting upright and head/neck flexed should be adopted in sarcopenic dysphagic patients, because this is the optimal posture to improve swallowing performances in dysphagic patients [202]. Moreover, postural adjustments significantly improve self-perceived difficulties in swallowing maneuvers [202]. In more detail, an upright 90° seated position should be maintained at least 30 min after eating to reduce the risk of inhalation of unswallowed food [18].

Swallowing compensatory maneuvers represent not only a short-term compensation to provide immediate benefits in bolus flow but also a specific rehabilitative strategy to improve swallowing functional training [81]. In more detail, supra- and super-supraglottic swallow, Mendelsohn’s maneuver and effortful swallow have been proposed to have a role in sarcopenic dysphagia.

Tongue-pressure resistance training (TPRT) is the most used strengthening exercise, and it has been proved to enable an improvement in hyoid bone movements, tongue pressure and width of the upper esophageal sphincter [203]. In 2020, Nagano et al. [178] reported an increased maximal tongue pressure in sarcopenic patients after 2-month physical and occupational therapy, without additional swallowing training. Therefore, these findings emphasized the role of a multitarget rehabilitative intervention focusing not only on swallowing training but including physical exercise combined with a rehabilitative nutrition approach to improve the overall well-being of older patients and consequent swallowing deficits related to a decrease of physical function and sarcopenia.

Lastly, compensatory strategies might include changes in the consistency of solid and/or liquid foods. In more detail, food texture represents one of the first therapeutic targets aimed at improving the safety and effectiveness of oral feeding and oral intake in dysphagic patients [81].

In accordance with the International Dysphagia Diet Standardization Initiative (IDDSI) framework [204], food texture can be categorized into eight different levels (0–7). The eight levels included both liquids (ranging from level 0 to level 4) and solids (ranging from level 3 to level 7). Therefore, levels 3 and 4 are characterized by a cross-over of both fluids and solids [204].

Based on these food texture levels, an IDDSI Functional Diet Scale [205] was introduced to standardize the food texture prescription based on the patient’s characteristics. Despite the great consensus received by the IDDSI worldwide, there is still a large heterogeneity of standards of diet texture and fluid modifications in the different countries [156].

Functional training might provide long-term benefits in patients suffering from dysphagia, such as lingual resistance exercises, considering the recent evidence supporting the strict relationship between sarcopenic dysphagia and tongue strength [206]. Given that resistance exercise requires specific and progressive training, a multidisciplinary approach should be provided, involving not only speech-language pathologists but also nurses, caregivers, and the patients themselves [85]. Moreover, specific exercises such as “shaker exercise” and “masako” (tongue hold) maneuver might improve swallowing physiology promoting muscles mechanics and bolus flow [207]. In this context, a meta-analysis performed by Carnaby-Mann et al. [208] assessed the effects of transcutaneous neuromuscular electrical stimulations for swallowing muscles, underlining the benefits of this treatment in improving swallowing performances. Moreover, strength deficits seem to be considered the main responsible for dysphagia in sarcopenic patients, suggesting intriguing implications of this treatment in elderly patients [206].

Despite several studies [209,210,211] underlining the positive effects of swallowing muscle training in swallowing function, dysphagia-related morbidities prevention and swallowing physiology improvement, there are only a few low-quality studies [188,189,191,192] that supported this rehabilitative intervention in patients with sarcopenic dysphagia.

Taken together, these findings highlight the effectiveness of a comprehensive oral rehabilitation approach in dysphagic patients, suggesting positive implications in sarcopenic dysphagia too. In contrast, there is still low evidence supporting oropharyngeal rehabilitation in a specific cohort of patients suffering from sarcopenic dysphagia, emphasizing the needing for clinical trials assessing this specific intervention in these patients.

## 8. Oral Health Management for Older Subjects

Populations across the world are ageing and the average life expectancy is rising in developed and developing countries [212]. On a global scale, the World Health Organization identified oral health as a major public health problem [213,214]. Among the oral diseases, caries and periodontal diseases are the most prevalent ones, even because the damage due to both periodontitis and caries is quite irreversible and so cumulative over the lifetime. Moreover, aging might affect both diseases directly, through aging of the immune system as well as impaired wound healing, and indirectly via physical and cognitive impairment as well as reduced access to care [215,216]. Indeed, the age-related decline in terms of physical performance and cognitive functions could influence older people’s ability to carry out an adequate oral hygiene, with a consequent higher prevalence of oral diseases (e.g., periodontal disease, caries, and oral mucosal inflammation) [129,217,218,219]. Moreover, as previously underlined, oral health status might be strictly linked to dysphagia and malnutrition with detrimental consequences in terms of the overall well-being of older adults.

In the literature, cognitive disorders have received growing attention for their possible link to oral diseases. In this context, studies have examined the effect of lower cognitive abilities on periodontal health, showing that reduced number of teeth, augmented alveolar bone loss, and increased pocket depth were associated with cognitive impairment [220,221]. If untreated, caries and periodontal diseases could lead to tooth loss, edentulism, reduction of the lower third of the face, poor esthetics, phonetic problems, loss of masticatory function, poor nutrition status, as well as loss of self-esteem, and reduced HRQoL [222,223]. Therefore, the treatment of caries, periodontitis, and replacement of teeth lost are the most used among the clinical approaches to improve masticatory function in older people and consequently might reduce the risk of malnutrition.

More in detail, the periodontal treatment aims to reduce the bacterial deposits with a reduction of the inflammatory response through the active therapy, which consists of full-mouth scaling (FMS) and full-mouth disinfection [224]. The scaling techniques allow the debridement of bacterial deposits coating the surface of the root, deep within the periodontal pocket. In addition, periodontal surgery is used when the depth of the periodontal pockets prevents adequate access for debridement. After causal therapy, a supportive periodontal therapy is employed to reduce the probability that the disease will flare up again, thus maintaining teeth without pain, excessive mobility, or persistent infection [225,226,227]. According to the American Academy of Periodontology, supportive periodontal therapy should include a periodontal re-evaluation and risk assessment, supragingival and subgingival removal of bacterial plaque and calculus, and re-treatment of any sites showing recurrent or persistent disease [225].

Frail and complex care needs elderly people suffered the most from oral dryness, which could lead to rapidly progressing caries and oral infections [113,228]. In this context, patients with xerostomia should be adequately treated to reduce dysgeusia, halitosis, burning mouth, oral pain, difficulty in chewing and swallowing, and speech impairment [120,121]. Salivary stimulation by means of systemic pharmacotherapies, such as pilocarpine, might be appropriate for use by patients with some degree of salivary gland function [115]. However, whereas the stimulation of saliva production could be effective, these drugs are associated with adverse effects and might be contraindicated in patients with existing chronic respiratory, cardiovascular and renal disease [229,230] in patients in whom drug therapy is contraindicated, non-pharmacological interventions, such as electrostimulation of the salivary glands, acupuncture or the application of low level laser therapy, have the potential to increase saliva production especially in patients with some residual salivary gland function [231].

The main goal of treatment of patients with co-morbidities or contraindications to pharmacological therapies remains the reduction of clinical symptoms to provide a short-term relief during daily hours [230]. Thus, the common therapy involves the topical application of salivary substitutes [231] or artificial saliva [232], including carboxymethylcellulose [233], herbal powder of Alcea digitata, and Malva sylvestris [234], and immunologically active saliva substitutes [235].

Replacement of teeth lost is conventionally addressed by replacing multiple missing teeth with prosthetic dental elements. Through the aid of partial or complete prosthesis (removable or fixed) it is possible to restore missing dental elements, both in patients who have the loss of some dental elements and in edentulous patients [236]. Techniques for replacing one or more missing teeth are removable dental prostheses, tooth and tooth-tissue supported, and fixed dental prostheses, tooth supported [237]. The use of complete removable dentures might induce clinical manifestations, such as stomatitis, traumatic ulcers, irritation-induced hyperplasia, and altered taste perception [237]. In this context, the implant-retained prostheses represent the new approach and a long-term therapeutic solution [238]. It is shown that prosthetic treatments could provide a better oral HRQoL in edentulous patients, and that the fixed implant-supported prostheses might also improve the patient satisfaction better than complete removable dentures treatment. These intriguing results might promote a significant improvement of masticatory function with possible implications in terms of oral intake and risk of malnutrition in older people.

It is important to highlight that the geriatric management should provide an interdisciplinary diagnostic and therapeutic process aimed at determining the psychophysical and functional problems of older people. Despite the significant progress in dental science and oral health prevention in recent years, chronic oral diseases are still common in older people [35].

Moreover, in geriatric subjects with serious illnesses and functional dependency, oral health problems are often underdiagnosed and untreated [239,240]. Increasing evidence also reveals significant interactions between oral health and general health that are unidirectional and often bidirectional [127,129,130,241,242]. It has been hypothesized that a strict correlation between oral health and quality of life, as reported by Hoeksema et al. [3] while assessing the oral health in community-living elderly, demonstrated that general health and HRQoL were higher in older people with remaining teeth and implant-supported dentures than the edentulous ones. Indeed, edentulous individuals with up to one denture were associated with higher risk of malnutrition, whereas in edentulous older persons with two complete dentures, a better nutritional status was observed [243].

Therefore, given that poor oral health has been recently identified as a determinant for malnutrition and sarcopenia [103], an adequate dental and oral screening might play a key role in a comprehensive management of older patients. Moreover, as suggested by the European Policy Recommendations on Oral Health in Older Adults [244], an adequate patient-tailored oral health rehabilitation program is crucial to prevent not only oral diseases but also malnutrition, particularly in the older people at high risk of sarcopenic dysphagia.

## 9. Study Limitations

We are aware that this study is not free from limitations. In particular, the narrative design severely limits the strength of the study results. However, it should be noted that considering the heterogeneity of pathological conditions and treatment assessed a systematic review was not possible according to the Cochrane Handbook for Systematic Review of Intervention (Ver, 6.1, 2020) [245].

## 10. Conclusions and Future Perspectives

This comprehensive review showed that there is a negative bond among sarcopenic dysphagia, malnutrition, and oral frailty in older people. These conditions share several risk factors some phenotypically overlapping features and should be adequately assessed and treated, particularly in the elderly.

A specific screening for sarcopenic dysphagia might be introduced in routine clinical practice for high-risk patients to promote early rehabilitative interventions preventing health-related consequences. Tailored treatment based on patient’s characteristics should be proposed aiming at targeting not only sarcopenic dysphagia consequences but also the etiological causes including oral frailty, malnutrition, and sarcopenia. Moreover, a comprehensive intervention should be proposed to promote the synergic effects of different therapies in frail elderly subjects.

Therefore, an adequate management of older people should include oropharyngeal rehabilitation, oral health treatment, and nutritional supplementation to counteract the age-related functional decline and to improve the quality of life.

Despite these considerations, future studies are warranted to provide strong evidence supporting a transdisciplinary approach to sarcopenic dysphagic elderly patients.

## Data Availability

Not applicable.

## References

[1] Abdullah B., Wolbring G. (2013). Analysis of newspaper coverage of active aging through the lens of the 2002 World Health Organization Active Ageing Report: A Policy Framework and the 2010 Toronto Charter for Physical Activity: A Global Call for Action. Int. J. Environ. Res. Public Health.

[2] Branca S., Bennati E., Ferlito L., Spallina G., Cardillo E., Malaguarnera M., Motta M. (2009). The health-care in the extreme longevity. Arch. Gerontol. Geriatr..

[3] Hoeksema A.R., Spoorenberg S., Peters L.L., Meijer H., Raghoebar G.M., Vissink A., Visser A. (2017). Elderly with remaining teeth report less frailty and better quality of life than edentulous elderly: A cross-sectional study. Oral. Dis..

[4] Kojima G., Liljas A.E.M., Iliffe S. (2019). Frailty syndrome: Implications and challenges for health care policy. Risk Manag. Healthc. Policy.

[5] De Sire A., Giachero A., De Santi S., Inglese K., Solaro C. (2021). Screening dysphagia risk in 534 older patients undergoing rehabilitation after total joint replacement: A cross-sectional study. Eur. J. Phys. Rehabil. Med..

[6] Fan Y., Ni S., Zhang H. (2021). Association between Healthy Eating Index-2015 total and component food scores with osteoporosis in middle-aged and older Americans: A cross-sectional study with U.S. National Health and Nutrition Examination Survey. Osteoporos. Int..

[7] Leigheb M., de Sire A., Colangelo M., Zagaria D., Grassi F.A., Rena O., Ferraro E. (2021). Sarcopenia Diagnosis: Reliability of the Ultrasound Assessment of the Tibialis Anterior Muscle as an Alternative Evaluation Tool. Diagnostics.

[8] Palmer K., Onder G., Cesari M. (2018). The geriatric condition of frailty. Eur. J. Intern. Med..

[9] Beard J.R., Officer A., de Carvalho I.A., Sadana R., Pot A.M., Michel J.P., Chatterji S. (2016). The World report on ageing and health: A policy framework for healthy ageing. Lancet.

[10] Hoogendijk E.O., Afilalo J., Ensrud K.E., Kowal P., Onder G., Fried L.P. (2019). Frailty: Implications for clinical practice and public health. Lancet.

[11] Di Monaco M., Castiglioni C., Bardesono F., Milano E., Massazza G. (2021). Sarcopenic obesity and function in women with subacute hip fracture: A short-term prospective study. Eur. J. Phys. Rehabil. Med..

[12] Cruz-Jentoft A.J., Baeyens J.P., Bauer J.M., Boirie Y., Cederholm T., Landi F., Zamboni M. (2010). Sarcopenia: European consensus on definition and diagnosis: Report of the European Working Group on Sarcopenia in Older People. Age Ageing.

[13] Mayhew A.J., Amog K., Phillips S., Parise G., McNicholas P.D., de Souza R.J., Raina P. (2019). The prevalence of sarcopenia in community-dwelling older adults, an exploration of differences between studies and within definitions: A systematic review and meta-analyses. Age Ageing.

[14] Agostini F., Bernetti A., Di Giacomo G., Viva M.G., Paoloni M., Mangone M., Masiero S. (2021). Rehabilitative Good Practices in the Treatment of Sarcopenia: A Narrative Review. Am. J. Phys. Med. Rehabil..

[15] Beaudart C., Rolland Y., Cruz-Jentoft A.J., Bauer J.M., Sieber C., Cooper C., Al-Daghri N., Araujo de Carvalho I., Bautmans I., Bernabei R. (2019). Assessment of Muscle Function and Physical Performance in Daily Clinical Practice: A position paper endorsed by the European Society for Clinical and Economic Aspects of Osteoporosis, Osteoarthritis and Musculoskeletal Diseases (ESCEO). Calcif. Tissue Int..

[16] Cha S., Kim W.S., Kim K.W., Han J.W., Jang H.C., Lim S., Paik N.J. (2019). Sarcopenia is an Independent Risk Factor for Dysphagia in Community-Dwelling Older Adults. Dysphagia.

[17] Maeda K., Takaki M., Akagi J. (2017). Decreased Skeletal Muscle Mass and Risk Factors of Sarcopenic Dysphagia: A Prospective Observational Cohort Study. J. Gerontol. A Biol. Sci. Med. Sci..

[18] Baijens L.W., Clavé P., Cras P., Ekberg O., Forster A., Kolb G.F., Walshe M. (2016). European Society for Swallowing Disorders—European Union Geriatric Medicine Society white paper: Oropharyngeal dysphagia as a geriatric syndrome. Clin. Interv. Aging.

[19] Dziewas R., Beck A.M., Clave P., Hamdy S., Heppner H.J., Langmore S.E., Wirth R. (2017). Recognizing the Importance of Dysphagia: Stumbling Blocks and Stepping Stones in the Twenty-First Century. Dysphagia.

[20] Wirth R., Dziewas R., Beck A.M., Clavé P., Hamdy S., Heppner H.J., Volkert D. (2016). Oropharyngeal dysphagia in older persons—from pathophysiology to adequate intervention: A review and summary of an international expert meeting. Clin. Interv. Aging.

[21] Kuroda Y., Kuroda R. (2012). Relationship between thinness and swallowing function in Japanese older adults: Implications for sarcopenic dysphagia. J. Am. Geriatr. Soc..

[22] Wakabayashi H. (2014). Presbyphagia and Sarcopenic Dysphagia: Association between Aging, Sarcopenia, and Deglutition Disorders. J. Frailty Aging.

[23] Invernizzi M., Baricich A., Riso S., Lippi L., Cisari C., de Sire A. (2019). Sarcopenic dysphagia: A narrative review. Clin. Cases Miner. Bone Metab..

[24] Mann T., Heuberger R., Wong H. (2013). The association between chewing and swallowing difficulties and nutritional status in older adults. Aust. Dent. J..

[25] N’Gom P.I., Woda A. (2002). Influence of impaired mastication on nutrition. J. Prosthet. Dent..

[26] Veldee M.S., Peth L.D. (1992). Can protein-calorie malnutrition cause dysphagia?. Dysphagia.

[27] Krishnamoorthy Y., Vijayageetha M., Kumar S.G., Rajaa S., Rehman T. (2018). Prevalence of malnutrition and its associated factors among elderly population in rural Puducherry using mini-nutritional assessment questionnaire. J. Fam. Med. Prim. Care.

[28] Lieu P.K., Chong M.S., Seshadri R. (2001). The impact of swallowing disorders in the elderly. Ann. Acad. Med. Singap..

[29] De Sire A., Baricich A., Ferrillo M., Migliario M., Cisari C., Invernizzi M. (2020). Buccal hemineglect: Is it useful to evaluate the differences between the two halves of the oral cavity for the multidisciplinary rehabilitative management of right brain stroke survivors? A cross-sectional study. Top. Stroke Rehabil..

[30] De Sire A., Invernizzi M., Ferrillo M., Gimigliano F., Baricich A., Cisari C., Migliario M. (2021). Functional status and oral health in patients with amyotrophic lateral sclerosis: A cross-sectional study. NeuroRehabilitation.

[31] Kloukos D., Roccuzzo A., Stähli A., Sculean A., Katsaros C., Salvi G.E. (2021). Effect of combined periodontal and orthodontic treatment of tilted molars and of teeth with intra-bony and furcation defects in stage-IV periodontitis patients: A systematic review. J. Clin. Periodontol..

[32] Toniazzo M.P., Amorim P.S., Muniz F., Weidlich P. (2018). Relationship of nutritional status and oral health in elderly: Systematic review with meta-analysis. Clin. Nutr..

[33] Lee K.H., Plassman B.L., Pan W., Wu B. (2016). Mediation Effect of Oral Hygiene on the Relationship Between Cognitive Function and Oral Health in Older Adults. J. Gerontol. Nurs..

[34] El Osta N., Hennequin M., Tubert-Jeannin S., Abboud Naaman N.B., El Osta L., Geahchan N. (2014). The pertinence of oral health indicators in nutritional studies in the elderly. Clin. Nutr..

[35] Petersen P.E., Yamamoto T. (2005). Improving the oral health of older people: The approach of the WHO Global Oral Health Programme. Community Dent. Oral Epidemiol..

[36] Lopez-Jornet P., Saura-Perez M., Llevat-Espinosa N. (2013). Effect of oral health dental state and risk of malnutrition in elderly people. Geriatr. Gerontol. Int..

[37] Kaiser M.J., Bauer J.M., Ramsch C., Uter W., Guigoz Y., Cederholm T., Sieber C.C. (2009). Validation of the Mini Nutritional Assessment short-form (MNA-SF): A practical tool for identification of nutritional status. J. Nutr. Health Aging.

[38] Vellas B., Villars H., Abellan G., Soto M.E., Rolland Y., Guigoz Y., Garry P. (2006). Overview of the MNA--Its history and challenges. J. Nutr. Health Aging.

[39] Rudnicka E., Napierała P., Podfigurna A., Męczekalski B., Smolarczyk R., Grymowicz M. (2020). The World Health Organization (WHO) approach to healthy ageing. Maturitas.

[40] Norman K., Haß U., Pirlich M. (2021). Malnutrition in Older Adults-Recent Advances and Remaining Challenges. Nutrients.

[41] Cederholm T., Bosaeus I., Barazzoni R., Bauer J., Van Gossum A., Klek S., Singer P. (2015). Diagnostic criteria for malnutrition—An ESPEN Consensus Statement. Clin. Nutr..

[42] Fleurke M., Voskuil D., Kolmer D. (2019). The role of the dietitian in the management of malnutrition in the elderly: A systematic review of current practices. Nutr. Diet..

[43] Cereda E., Pedrolli C., Klersy C., Bonardi C., Quarleri L., Cappello S., Caccialanza R. (2016). Nutritional status in older persons according to healthcare setting: A systematic review and meta-analysis of prevalence data using MNA(^®^). Clin. Nutr..

[44] Wojzischke J., van Wijngaarden J., van den Berg C., Cetinyurek-Yavuz A., Diekmann R., Luiking Y., Bauer J. (2020). Nutritional status and functionality in geriatric rehabilitation patients: A systematic review and meta-analysis. Eur. Geriatr. Med..

[45] Landi F., Calvani R., Tosato M., Martone A.M., Ortolani E., Savera G., Marzetti E. (2016). Anorexia of Aging: Risk Factors, Consequences, and Potential Treatments. Nutrients.

[46] Naruishi K., Nishikawa Y. (2018). Swallowing impairment is a significant factor for predicting life prognosis of elderly at the end of life. Aging Clin. Exp. Res..

[47] Hägglund P., Fält A., Hägg M., Wester P., Levring Jäghagen E. (2019). Swallowing dysfunction as risk factor for undernutrition in older people admitted to Swedish short-term care: A cross-sectional study. Aging Clin. Exp. Res..

[48] Nishioka S., Okamoto T., Takayama M., Urushihara M., Watanabe M., Kiriya Y., Kageyama N. (2017). Malnutrition risk predicts recovery of full oral intake among older adult stroke patients undergoing enteral nutrition: Secondary analysis of a multicentre survey (the APPLE study). Clin. Nutr..

[49] Saarela R.K., Soini H., Hiltunen K., Muurinen S., Suominen M., Pitkälä K. (2014). Dentition status, malnutrition and mortality among older service housing residents. J. Nutr. Health Aging.

[50] Anastassiadou V., Heath M.R. (2002). Food choices and eating difficulty among elderly edentate patients in Greece. Gerodontology.

[51] Joshipura K.J., Willett W.C., Douglass C.W. (1996). The impact of edentulousness on food and nutrient intake. J. Am. Dent. Assoc..

[52] O’Connor J.P., Milledge K.L., O’Leary F., Cumming R., Eberhard J., Hirani V. (2020). Poor dietary intake of nutrients and food groups are associated with increased risk of periodontal disease among community-dwelling older adults: A systematic literature review. Nutr. Rev..

[53] Kotsakis G.A., Chrepa V., Shivappa N., Wirth M., Hébert J., Koyanagi A., Tyrovolas S. (2018). Diet-borne systemic inflammation is associated with prevalent tooth loss. Clin. Nutr..

[54] Van der Putten G.J., Vanobbergen J., De Visschere L., Schols J., de Baat C. (2009). Association of some specific nutrient deficiencies with periodontal disease in elderly people: A systematic literature review. Nutrition.

[55] Vellas B., Guigoz Y., Garry P.J., Nourhashemi F., Bennahum D., Lauque S., Albarede J.L. (1999). The Mini Nutritional Assessment (MNA) and its use in grading the nutritional state of elderly patients. Nutrition.

[56] Chapple I.L.C., Mealey B.L., Van Dyke T.E., Bartold P.M., Dommisch H., Eickholz P., Yoshie H. (2018). Periodontal health and gingival diseases and conditions on an intact and a reduced periodontium: Consensus report of workgroup 1 of the 2017 World Workshop on the Classification of Periodontal and Peri-Implant Diseases and Conditions. J. Periodontol..

[57] Ferguson M., Capra S., Bauer J., Banks M. (1999). Development of a valid and reliable malnutrition screening tool for adult acute hospital patients. Nutrition.

[58] Keller H.H., Goy R., Kane S.L. (2005). Validity and reliability of SCREEN II (Seniors in the community: Risk evaluation for eating and nutrition, Version II). Eur. J. Clin. Nutr..

[59] Stratton R.J., King C.L., Stroud M.A., Jackson A.A., Elia M. (2006). ‘Malnutrition Universal Screening Tool’ predicts mortality and length of hospital stay in acutely ill elderly. Br. J. Nutr..

[60] Wilson M.M., Thomas D.R., Rubenstein L.Z., Chibnall J.T., Anderson S., Baxi A., Morley J.E. (2005). Appetite assessment: Simple appetite questionnaire predicts weight loss in community-dwelling adults and nursing home residents. Am. J. Clin. Nutr..

[61] Kondrup J., Rasmussen H.H., Hamberg O., Stanga Z. (2003). Nutritional risk screening (NRS 2002): A new method based on an analysis of controlled clinical trials. Clin. Nutr..

[62] Bai A.V., Agostini F., Bernetti A., Mangone M., Fidenzi G., D’Urzo R., Masiero S. (2021). State of the evidence about rehabilitation interventions in patients with dysphagia. Eur. J. Phys. Rehabil. Med..

[63] Eroğlu A.G. (2019). Malnutrition and the heart. Turk. Pediatri. Ars..

[64] Raposeiras Roubín S., Abu Assi E., Cespón Fernandez M., Barreiro Pardal C., Lizancos Castro A., Parada J.A., Íñiguez Romo A. (2020). Prevalence and Prognostic Significance of Malnutrition in Patients with Acute Coronary Syndrome. J. Am. Coll. Cardiol..

[65] Wells J.C., Sawaya A.L., Wibaek R., Mwangome M., Poullas M.S., Yajnik C.S., Demaio A. (2020). The double burden of malnutrition: Aetiological pathways and consequences for health. Lancet.

[66] Elmadfa I., Meyer A.L. (2019). The Role of the Status of Selected Micronutrients in Shaping the Immune Function. Endocr. Metab. Immune Disord. Drug Targets.

[67] Cantorna M.T., McDaniel K., Bora S., Chen J., James J. (2014). Vitamin D, immune regulation, the microbiota, and inflammatory bowel disease. Exp. Biol. Med..

[68] Andrès E., Loukili N.H., Noel E., Kaltenbach G., Abdelgheni M.B., Perrin A.E., Blicklé J.F. (2004). Vitamin B12 (cobalamin) deficiency in elderly patients. Cmaj.

[69] Cunha D.F., Cunha S.F., Unamuno M.R., Vannucchi H. (2001). Serum levels assessment of vitamin A, E, C, B2 and carotenoids in malnourished and non-malnourished hospitalized elderly patients. Clin. Nutr..

[70] Spinneker A., Sola R., Lemmen V., Castillo M.J., Pietrzik K., González-Gross M. (2007). Vitamin B6 status, deficiency and its consequences—An overview. Nutr. Hosp..

[71] Vaquero M.P. (2002). Magnesium and trace elements in the elderly: Intake, status and recommendations. J. Nutr. Health Aging.

[72] Carmel R. (2008). Nutritional anemias and the elderly. Semin. Hematol..

[73] Cruz-Jentoft A.J., Kiesswetter E., Drey M., Sieber C.C. (2017). Nutrition, frailty, and sarcopenia. Aging Clin. Exp. Res..

[74] Rizzoli R. (2014). Nutritional aspects of bone health. Best Pract. Res. Clin. Endocrinol. Metab..

[75] Gaffney-Stomberg E., Insogna K.L., Rodriguez N.R., Kerstetter J.E. (2009). Increasing dietary protein requirements in elderly people for optimal muscle and bone health. J. Am. Geriatr. Soc..

[76] Rizzoli R. (2018). Postmenopausal osteoporosis: Assessment and management. Best Pract. Res. Clin. Endocrinol. Metab..

[77] Bikle D.D., Tahimic C., Chang W., Wang Y., Philippou A., Barton E.R. (2015). Role of IGF-I signaling in muscle bone interactions. Bone.

[78] De Sire A., Invernizzi M., Baricich A., Lippi L., Ammendolia A., Grassi F.A., Leigheb M. (2021). Optimization of transdisciplinary management of elderly with femur proximal extremity fracture: A patient-tailored plan from orthopaedics to rehabilitation. World J. Orthop..

[79] Marzetti E., Calvani R., Tosato M., Cesari M., Di Bari M., Cherubini A., Broccatelli M., Savera G., D’Elia M., Pahor M. (2017). Physical activity and exercise as countermeasures to physical frailty and sarcopenia. Aging Clin. Exp. Res..

[80] Namasivayam A.M., Steele C.M. (2015). Malnutrition and Dysphagia in long-term care: A systematic review. J. Nutr. Gerontol. Geriatr..

[81] Sura L., Madhavan A., Carnaby G., Crary M.A. (2012). Dysphagia in the elderly: Management and nutritional considerations. Clin. Interv. Aging.

[82] Damanti S., Azzolino D., Roncaglione C., Arosio B., Rossi P., Cesari M. (2019). Efficacy of Nutritional Interventions as Stand-Alone or Synergistic Treatments with Exercise for the Management of Sarcopenia. Nutrients.

[83] Logrippo S., Ricci G., Sestili M., Cespi M., Ferrara L., Palmieri G.F., Blasi P. (2017). Oral drug therapy in elderly with dysphagia: Between a rock and a hard place!. Clin. Interv. Aging.

[84] Siebens H., Trupe E., Siebens A., Cook F., Anshen S., Hanauer R., Oster G. (1986). Correlates and consequences of eating dependency in institutionalized elderly. J. Am. Geriatr. Soc..

[85] Dellis S., Papadopoulou S., Krikonis K., Zigras F. (2018). Sarcopenic Dysphagia. A Narrative Review. J. Frailty Sarcopenia Falls.

[86] Feng X., Todd T., Lintzenich C.R., Ding J., Carr J.J., Ge Y., Butler S.G. (2013). Aging-related geniohyoid muscle atrophy is related to aspiration status in healthy older adults. J. Gerontol. A Biol. Sci. Med. Sci..

[87] Nakao Y., Uchiyama Y., Honda K., Yamashita T., Saito S., Domen K. (2021). Age-related composition changes in swallowing-related muscles: A Dixon MRI study. Aging Clin. Exp. Res..

[88] Miyashita T., Kikutani T., Nagashima K., Igarashi K., Tamura F. (2020). The effects of sarcopenic dysphagia on the dynamics of swallowing organs observed on videofluoroscopic swallowing studies. J. Oral Rehabil..

[89] Kunieda K., Fujishima I., Wakabayashi H., Ohno T., Shigematsu T., Itoda M., Ogawa S. (2021). Relationship Between Tongue Pressure and Pharyngeal Function Assessed Using High-Resolution Manometry in Older Dysphagia Patients with Sarcopenia: A Pilot Study. Dysphagia.

[90] Nawaz S., Tulunay-Ugur O.E. (2018). Dysphagia in the Older Patient. Otolaryngol. Clin. N. Am..

[91] Belafsky P.C., Mouadeb D.A., Rees C.J., Pryor J.C., Postma G.N., Allen J., Leonard R.J. (2008). Validity and reliability of the Eating Assessment Tool (EAT-10). Ann. Otol. Rhinol. Laryngol..

[92] Cheney D.M., Siddiqui M.T., Litts J.K., Kuhn M.A., Belafsky P.C. (2015). The Ability of the 10-Item Eating Assessment Tool (EAT-10) to Predict Aspiration Risk in Persons with Dysphagia. Ann. Otol. Rhinol. Laryngol..

[93] Tohara H., Saitoh E., Mays K.A., Kuhlemeier K., Palmer J.B. (2003). Three tests for predicting aspiration without videofluorography. Dysphagia.

[94] Yagi N., Oku Y., Nagami S., Yamagata Y., Kayashita J., Ishikawa A., Takahashi R. (2017). Inappropriate Timing of Swallow in the Respiratory Cycle Causes Breathing-Swallowing Discoordination. Front. Physiol..

[95] Audag N., Goubau C., Toussaint M., Reychler G. (2019). Screening and evaluation tools of dysphagia in adults with neuromuscular diseases: A systematic review. Ther. Adv. Chronic. Dis..

[96] Crary M.A., Mann G.D., Groher M.E. (2005). Initial psychometric assessment of a functional oral intake scale for dysphagia in stroke patients. Arch. Phys. Med. Rehabil..

[97] Kunieda K., Ohno T., Fujishima I., Hojo K., Morita T. (2013). Reliability and validity of a tool to measure the severity of dysphagia: The Food Intake LEVEL Scale. J. Pain Symptom Manag..

[98] Giraldo-Cadavid L.F., Pantoja J.A., Forero Y., Gutierrez H.M., Bastidas A. (2019). Aspiration in the Fiberoptic Endoscopic Evaluation of Swallowing Associated with an Increased Risk of Mortality in a Cohort of Patients Suspected of Oropharyngeal Dysphagia. Dysphagia.

[99] Budtz-Jørgensen E., Chung J.P., Mojon P. (2000). Successful aging—The case for prosthetic therapy. J. Public Health Dent..

[100] Avlund K., Schultz-Larsen K., Christiansen N., Holm-Pedersen P. (2011). Number of Teeth and Fatigue in Older Adults. J. Am. Geriatr. Soc..

[101] Coleman P. (2002). Improving oral health care for the frail elderly: A review of widespread problems and best practices. Geriatr. Nurs..

[102] Iwasaki M., Taylor G.W., Manz M.C., Yoshihara A., Sato M., Muramatsu K., Miyazaki H. (2014). Oral health status: Relationship to nutrient and food intake among 80-year-old Japanese adults. Community Dent. Oral Epidemiol..

[103] Azzolino D., Passarelli P.C., De Angelis P., Piccirillo G.B., D’Addona A., Cesari M. (2019). Poor Oral Health as a Determinant of Malnutrition and Sarcopenia. Nutrients.

[104] Veronese N., Punzi L., Sieber C., Bauer J., Reginster J.Y., Maggi S., the Task Finish Group on “Arthritis” of the European Geriatric Medicine Society (2018). Sarcopenic osteoarthritis: A new entity in geriatric medicine?. Eur. Geriatr. Med..

[105] Hussein S., Kantawalla R.F., Dickie S., Suarez-Durall P., Enciso R., Mulligan R. (2021). Association of Oral Health and Mini Nutritional Assessment in Older Adults: A Systematic Review with Meta-analyses. J. Prosthodont. Res..

[106] Iwasaki M., Motokawa K., Watanabe Y., Shirobe M., Inagaki H., Edahiro A., Awata S. (2020). Association between Oral Frailty and Nutritional Status among Community-Dwelling Older Adults: The Takashimadaira Study. J. Nutr. Health Aging.

[107] Lamy M., Mojon P., Kalykakis G., Legrand R., Butz-Jorgensen E. (1999). Oral status and nutrition in the institutionalized elderly. J. Dent..

[108] Anil S., Vellappally S., Hashem M., Preethanath R.S., Patil S., Samaranayake L.P. (2016). Xerostomia in geriatric patients: A burgeoning global concern. J. Investig. Clin. Dent..

[109] Liu B., Dion M.R., Jurasic M.M., Gibson G., Jones J.A. (2012). Xerostomia and salivary hypofunction in vulnerable elders: Prevalence and etiology. Oral Surg. Oral Med. Oral Pathol. Oral Radiol..

[110] Ouanounou A. (2016). Xerostomia in the Geriatric Patient: Causes, Oral Manifestations, and Treatment. Compend. Contin. Educ. Dent..

[111] Thomson W.M., Chalmers J.M., Spencer A.J., Williams S.M. (1999). The Xerostomia Inventory: A multi-item approach to measuring dry mouth. Community Dent. Health.

[112] Nederfors T., Isaksson R., Mörnstad H., Dahlöf C. (1997). Prevalence of perceived symptoms of dry mouth in an adult Swedish population--relation to age, sex and pharmacotherapy. Community Dent. Oral. Epidemiol..

[113] Aliko A., Wolff A., Dawes C., Aframian D., Proctor G., Ekström J., Vissink A. (2015). World Workshop on Oral Medicine VI: Clinical implications of medication-induced salivary gland dysfunction. Oral Surg. Oral Med. Oral Pathol. Oral Radiol..

[114] Von Bültzingslöwen I., Sollecito T.P., Fox P.C., Daniels T., Jonsson R., Lockhart P.B., Schiødt M. (2007). Salivary dysfunction associated with systemic diseases: Systematic review and clinical management recommendations. Oral Surg. Oral Med. Oral Pathol. Oral Radiol. Endodontol..

[115] Porter S.R., Scully C., Hegarty A.M. (2004). An update of the etiology and management of xerostomia. Oral Surg. Oral Med. Oral Pathol. Oral Radiol. Endodontol..

[116] Napeñas J.J., Brennan M.T., Fox P.C. (2009). Diagnosis and treatment of xerostomia (dry mouth). Odontology.

[117] Carrozzo M. (2008). Oral diseases associated with hepatitis C virus infection. Part 1. sialadenitis and salivary glands lymphoma. Oral Dis..

[118] Humphrey S.P., Williamson R.T. (2001). A review of saliva: Normal composition, flow, and function. J. Prosthet. Dent..

[119] Salles C., Chagnon M.-C., Feron G., Guichard E., Laboure H., Morzel M., Yven C. (2011). In-mouth mechanisms leading to flavor release and perception. Crit. Rev. Food Sci. Nutr..

[120] Han P., Suarez-Durall P., Mulligan R. (2015). Dry mouth: A critical topic for older adult patients. J. Prosthodont. Res..

[121] Muñoz-González C., Vandenberghe-Descamps M., Feron G., Canon F., Labouré H., Sulmont-Rossé C. (2017). Association between Salivary Hypofunction and Food Consumption in the Elderlies. A Systematic Literature Review. J. Nutr. Health Aging.

[122] Di Stasio D., Lauritano D., Minervini G., Paparella R.S., Petruzzi M., Romano A., Lucchese A. (2018). Management of denture stomatitis: A narrative review. J. Biol. Regul. Homeost. Agents.

[123] Kaplan I., Zuk-Paz L., Wolff A. (2008). Association between salivary flow rates, oral symptoms, and oral mucosal status. Oral Surg. Oral Med. Oral Pathol. Oral Radiol. Endodontol..

[124] Plemons J.M., Al-Hashimi I., Marek C.L. (2014). Managing xerostomia and salivary gland hypofunction: Executive summary of a report from the American Dental Association Council on Scientific Affairs. J. Am. Dent. Assoc..

[125] Könönen E., Gursoy M., Gursoy U.K. (2019). Periodontitis: A Multifaceted Disease of Tooth-Supporting Tissues. J. Clin. Med..

[126] Sanz M., van Winkelhoff A.J. (2011). Periodontal infections: Understanding the complexity--consensus of the Seventh European Workshop on Periodontology. J. Clin. Periodontol..

[127] Ferrillo M., Migliario M., Roccuzzo A., Molinero-Mourelle P., Falcicchio G., Umano G.R., de Sire A. (2021). Periodontal Disease and Vitamin D Deficiency in Pregnant Women: Which Correlation with Preterm and Low-Weight Birth?. J. Clin. Med..

[128] Aimetti M., Romano F., Nessi F. (2007). Microbiologic Analysis of Periodontal Pockets and Carotid Atheromatous Plaques in Advanced Chronic Periodontitis Patients. J. Periodontol..

[129] Carrizales-Sepúlveda E.F., Ordaz-Farías A., Vera-Pineda R., Flores-Ramírez R. (2018). Periodontal Disease, Systemic Inflammation and the Risk of Cardiovascular Disease. Heart Lung Circ..

[130] Li W., Xu J., Zhang R., Li Y., Wang J., Zhang X., Lin L. (2021). Is periodontal disease a risk indicator for colorectal cancer? A systematic review and meta-analysis. J. Clin. Periodontol..

[131] Chandra R.K. (1997). Nutrition and the immune system: An introduction. Am. J. Clin. Nutr..

[132] Cunningham-Rundles S. (1998). Analytical Methods for Evaluation of Immune Response in Nutrient Intervention. Nutr. Rev..

[133] Dommisch H., Kuzmanova D., Jönsson D., Grant M., Chapple I. (2018). Effect of micronutrient malnutrition on periodontal disease and periodontal therapy. Periodontol. 2000.

[134] Džopalić T., Božić-Nedeljković B., Jurišić V. (2021). The role of vitamin A and vitamin D in modulation of the immune response with a focus on innate lymphoid cells. Cent. Eur. J. Immunol..

[135] Meydani S.N., Meydani M., Blumberg J.B., Leka L.S., Siber G., Loszewski R., Stollar B.D. (1997). Vitamin E supplementation and in vivo immune response in healthy elderly subjects. A randomized controlled trial. JAMA.

[136] Boyd L.D., Madden T.E. (2003). Nutrition, infection, and periodontal disease. Dent. Clin. N. Am..

[137] Hatta K., Ikebe K. (2021). Association between oral health and sarcopenia: A literature review. J. Prosthodont. Res..

[138] Chang H.R., Bistrian B. (1998). The role of cytokines in the catabolic consequences of infection and injury. JPEN J. Parenter. Enteral Nutr..

[139] Visser M., Pahor M., Taaffe D.R., Goodpaster B.H., Simonsick E.M., Newman A.B., Harris T.B. (2002). Relationship of Interleukin-6 and Tumor Necrosis Factor-α With Muscle Mass and Muscle Strength in Elderly Men and Women: The Health ABC Study. J. Gerontol. Ser. A.

[140] Payette H., Roubenoff R., Jacques P.F., Dinarello C.A., Wilson P.W.F., Abad L.W., Harris T. (2003). Insulin-like growth factor-1 and interleukin 6 predict sarcopenia in very old community-living men and women: The Framingham Heart Study. J. Am. Geriatr. Soc..

[141] Shiraishi A., Yoshimura Y., Wakabayashi H., Tsuji Y. (2018). Prevalence of stroke-related sarcopenia and its association with poor oral status in post-acute stroke patients: Implications for oral sarcopenia. Clin. Nutr..

[142] Ortega O., Parra C., Zarcero S., Nart J., Sakwinska O., Clavé P. (2014). Oral health in older patients with oropharyngeal dysphagia. Age Ageing.

[143] Poisson P., Laffond T., Campos S., Dupuis V., Bourdel-Marchasson I. (2016). Relationships between oral health, dysphagia and undernutrition in hospitalised elderly patients. Gerodontology.

[144] Osterberg T., Tsuga K., Rothenberg E., Carlsson G.E., Steen B. (2002). Masticatory ability in 80-year-old subjects and its relation to intake of energy, nutrients and food items. Gerodontology.

[145] Hämäläinen P., Rantanen T., Keskinen M., Meurman J.H. (2004). Oral health status and change in handgrip strength over a 5-year period in 80-year-old people. Gerodontology.

[146] Sakai K., Nakayama E., Tohara H., Maeda T., Sugimoto M., Takehisa T., Ueda K. (2017). Tongue Strength is Associated with Grip Strength and Nutritional Status in Older Adult Inpatients of a Rehabilitation Hospital. Dysphagia.

[147] Yamaguchi K., Tohara H., Hara K., Nakane A., Kajisa E., Yoshimi K., Minakuchi S. (2018). Relationship of aging, skeletal muscle mass, and tooth loss with masseter muscle thickness. BMC Geriatr..

[148] Picca A., Calvani R., Manes-Gravina E., Spaziani L., Landi F., Bernabei R., Marzetti E. (2017). Bone-Muscle Crosstalk: Unraveling New Therapeutic Targets for Osteoporosis. Curr. Pharm. Des..

[149] Contaldo M., Itro A., Lajolo C., Gioco G., Inchingolo F., Serpico R. (2020). Overview on Osteoporosis, Periodontitis and Oral Dysbiosis: The Emerging Role of Oral Microbiota. Appl. Sci..

[150] Fujishima I., Fujiu-Kurachi M., Arai H., Hyodo M., Kagaya H., Maeda K., Yoshimura Y. (2019). Sarcopenia and dysphagia: Position paper by four professional organizations. Geriatr. Gerontol. Int..

[151] Pizzoferrato M., de Sire R., Ingravalle F., Mentella M.C., Petito V., Martone A.M., Gasbarrini A. (2019). Characterization of Sarcopenia in an IBD Population Attending an Italian Gastroenterology Tertiary Center. Nutrients.

[152] Larsson L., Degens H., Li M., Salviati L., Lee Y.i., Thompson W., Sandri M. (2019). Sarcopenia: Aging-Related Loss of Muscle Mass and Function. Physiol. Rev..

[153] De Carvalho F.G., Justice J.N., Freitas E.C., Kershaw E.E., Sparks L.M. (2019). Adipose Tissue Quality in Aging: How Structural and Functional Aspects of Adipose Tissue Impact Skeletal Muscle Quality. Nutrients.

[154] De Sire A., de Sire R., Petito V., Masi L., Cisari C., Gasbarrini A., Invernizzi M. (2020). Gut-Joint Axis: The Role of Physical Exercise on Gut Microbiota Modulation in Older People with Osteoarthritis. Nutrients.

[155] Nardone O.M., de Sire R., Petito V., Testa A., Villani G., Scaldaferri F., Castiglione F. (2021). Inflammatory Bowel Diseases and Sarcopenia: The Role of Inflammation and Gut Microbiota in the Development of Muscle Failure. Front. Immunol..

[156] Azzolino D., Damanti S., Bertagnoli L., Lucchi T., Cesari M. (2019). Sarcopenia and swallowing disorders in older people. Aging Clin. Exp. Res..

[157] Molfenter S.M., Amin M.R., Branski R.C., Brumm J.D., Hagiwara M., Roof S.A., Lazarus C.L. (2015). Age-Related Changes in Pharyngeal Lumen Size: A Retrospective MRI Analysis. Dysphagia.

[158] Suzuki M., Koyama S., Kimura Y., Ishiyama D., Ohji S., Otobe Y., Yamada M. (2019). Relationship between tongue muscle quality and swallowing speed in community-dwelling older women. Aging Clin. Exp. Res..

[159] Mizuno S., Wakabayashi H., Wada F. (2022). Rehabilitation nutrition for individuals with frailty, disability, sarcopenic dysphagia, or sarcopenic respiratory disability. Curr. Opin. Clin. Nutr. Metab. Care.

[160] Ogawa N., Mori T., Fujishima I., Wakabayashi H., Itoda M., Kunieda K., Ogawa S. (2017). Ultrasonography to Measure Swallowing Muscle Mass and Quality in Older Patients with Sarcopenic Dysphagia. J. Am. Med. Dir. Assoc..

[161] Martone A.M., Marzetti E., Calvani R., Picca A., Tosato M., Santoro L., Landi F. (2017). Exercise and Protein Intake: A Synergistic Approach against Sarcopenia. Biomed. Res. Int..

[162] Matsuo H., Yoshimura Y., Fujita S., Maeno Y. (2020). Dysphagia is associated with poor physical function in patients with acute heart failure: A prospective cohort study. Aging Clin. Exp. Res..

[163] Zhao W.T., Yang M., Wu H.M., Yang L., Zhang X.M., Huang Y. (2018). Systematic Review and Meta-Analysis of the Association between Sarcopenia and Dysphagia. J. Nutr. Health Aging.

[164] Mori T., Fujishima I., Wakabayashi H., Oshima F., Itoda M., Kunieda K., Ogawa S. (2017). Development, reliability, and validity of a diagnostic algorithm for sarcopenic dysphagia. JCSM Clin. Rep..

[165] Sporns P.B., Muhle P., Hanning U., Suntrup-Krueger S., Schwindt W., Eversmann J., Dziewas R. (2017). Atrophy of Swallowing Muscles Is Associated With Severity of Dysphagia and Age in Patients With Acute Stroke. J. Am. Med. Dir. Assoc..

[166] Can B., İsmagulova N., Enver N., Tufan A., Cinel İ. (2021). Sarcopenic dysphagia following COVID-19 infection: A new danger. Nutr. Clin. Pract..

[167] Brodsky M.B., Gilbert R.J. (2020). The Long-Term Effects of COVID-19 on Dysphagia Evaluation and Treatment. Arch. Phys. Med. Rehabil..

[168] Agostini F., Mangone M., Ruiu P., Paolucci T., Santilli V., Bernetti A. (2021). Rehabilitation setting during and after Covid-19: An overview on recommendations. J. Rehabil. Med..

[169] Roberts S., Collins P., Rattray M. (2021). Identifying and Managing Malnutrition, Frailty and Sarcopenia in the Community: A Narrative Review. Nutrients.

[170] Kiss N., Bauer J., Boltong A., Brown T., Isenring L., Loeliger J., Findlay M. (2020). Awareness, perceptions and practices regarding cancer-related malnutrition and sarcopenia: A survey of cancer clinicians. Support. Care Cancer.

[171] Vellas B., Fielding R.A., Bens C., Bernabei R., Cawthon P.M., Cederholm T., Cesari M. (2018). Implications of ICD-10 for Sarcopenia Clinical Practice and Clinical Trials: Report by the International Conference on Frailty and Sarcopenia Research Task Force. J. Frailty Aging.

[172] Nagano A., Nishioka S., Wakabayashi H. (2019). Rehabilitation Nutrition for Iatrogenic Sarcopenia and Sarcopenic Dysphagia. J. Nutr. Health Aging.

[173] Chen K.-C., Jeng Y., Wu W.-T., Wang T.-G., Han D.-S., Özçakar L., Chang K.-V. (2021). Sarcopenic Dysphagia: A Narrative Review from Diagnosis to Intervention. Nutrients.

[174] Shimizu A., Fujishima I., Maeda K., Wakabayashi H., Nishioka S., Ohno T. (2021). The Japanese Working Group on Sarcopenic, D. Nutritional Management Enhances the Recovery of Swallowing Ability in Older Patients with Sarcopenic Dysphagia. Nutrients.

[175] Nagano A., Maeda K., Shimizu A., Nagami S., Takigawa N., Ueshima J., Suenaga M. (2020). Association of Sarcopenic Dysphagia with Underlying Sarcopenia Following Hip Fracture Surgery in Older Women. Nutrients.

[176] Volkert D., Beck A.M., Cederholm T., Cruz-Jentoft A., Goisser S., Hooper L., Bischoff S.C. (2019). ESPEN guideline on clinical nutrition and hydration in geriatrics. Clin. Nutr..

[177] Bauer J., Biolo G., Cederholm T., Cesari M., Cruz-Jentoft A.J., Morley J.E., Boirie Y. (2013). Evidence-based recommendations for optimal dietary protein intake in older people: A position paper from the PROT-AGE Study Group. J. Am. Med. Dir. Assoc..

[178] Nagano A., Maeda K., Koike M., Murotani K., Ueshima J., Shimizu A., Mori N. (2020). Effects of Physical Rehabilitation and Nutritional Intake Management on Improvement in Tongue Strength in Sarcopenic Patients. Nutrients.

[179] Hudson H.M., Daubert C.R., Mills R.H. (2000). The interdependency of protein-energy malnutrition, aging, and dysphagia. Dysphagia.

[180] Harvey S.E., Parrott F., Harrison D.A., Bear D.E., Segaran E., Beale R., Rowan K.M. (2014). Trial of the Route of Early Nutritional Support in Critically Ill Adults. N. Engl. J. Med..

[181] Lewis S.R., Schofield-Robinson O.J., Alderson P., Smith A.F. (2018). Enteral versus parenteral nutrition and enteral versus a combination of enteral and parenteral nutrition for adults in the intensive care unit. Cochrane Database Syst. Rev..

[182] Cichero J.A.Y. (2018). Age-Related Changes to Eating and Swallowing Impact Frailty: Aspiration, Choking Risk, Modified Food Texture and Autonomy of Choice. Geriatrics.

[183] Johnson M.A., Kimlin M.G. (2006). Vitamin D, Aging, and the 2005 Dietary Guidelines for Americans. Nutr. Rev..

[184] Invernizzi M., de Sire A., D’Andrea F., Carrera D., Renò F., Migliaccio S., Cisari C. (2019). Effects of essential amino acid supplementation and rehabilitation on functioning in hip fracture patients: A pilot randomized controlled trial. Aging Clin. Exp. Res..

[185] Iolascon G., Mauro G.L., Fiore P., Cisari C., Benedetti M.G., Panella L., Gimigliano F. (2018). Can vitamin D deficiency influence muscle performance in postmenopausal women? A multicenter retrospective study. Eur. J. Phys. Rehabil. Med..

[186] Gimigliano F., Moretti A., de Sire A., Calafiore D., Iolascon G. (2018). The combination of vitamin D deficiency and overweight affects muscle mass and function in older post-menopausal women. Aging Clin. Exp. Res..

[187] Hashida N., Shamoto H., Maeda K., Wakabayashi H., Suzuki M., Fujii T. (2017). Rehabilitation and nutritional support for sarcopenic dysphagia and tongue atrophy after glossectomy: A case report. Nutrition.

[188] Yamada Y., Shamoto H., Maeda K., Wakabayashi H. (2018). Home-based Combined Therapy with Rehabilitation and Aggressive Nutrition Management for a Parkinson’s Disease Patient with Sarcopenic Dysphagia: A Case Report. Prog. Rehabil. Med..

[189] Wakabayashi H., Uwano R. (2016). Rehabilitation Nutrition for Possible Sarcopenic Dysphagia After Lung Cancer Surgery: A Case Report. Am. J. Phys. Med. Rehabil..

[190] Borda M.G., Venegas-Sanabria L.C., Puentes-Leal G.A., Garcia-Cifuentes E., Chavarro-Carvajal D.A., Cano C.A. (2017). Oropharyngeal dysphagia in older adults: The well-known tale. Geriatr. Gerontol. Int..

[191] Paolucci T., Cardarola A., Colonnelli P., Ferracuti G., Gonnella R., Murgia M., Mangone M. (2020). Give me a kiss! An integrative rehabilitative training program with motor imagery and mirror therapy for recovery of facial palsy. Eur. J. Phys. Rehabil. Med..

[192] Maeda K., Akagi J. (2016). Treatment of Sarcopenic Dysphagia with Rehabilitation and Nutritional Support: A Comprehensive Approach. J. Acad. Nutr. Diet..

[193] Nakayama E., Tohara H., Sato M., Hino H., Sakai M., Nagashima Y., Ooshima M. (2019). Time Course and Recovery of the Movements of Hyoid Bone and Thyroid Cartilage During Swallowing in a Patient with Sarcopenic Dysphagia. Am. J. Phys. Med. Rehabil..

[194] Wakabayashi H., Takahashi R., Murakami T. (2019). The Prevalence and Prognosis of Sarcopenic Dysphagia in Patients Who Require Dysphagia Rehabilitation. J. Nutr. Health Aging.

[195] Yamada M., Kimura Y., Ishiyama D., Nishio N., Otobe Y., Tanaka T., Arai H. (2019). Synergistic effect of bodyweight resistance exercise and protein supplementation on skeletal muscle in sarcopenic or dynapenic older adults. Geriatr. Gerontol. Int..

[196] Yoshimura Y., Uchida K., Jeong S., Yamaga M. (2016). Effects of Nutritional Supplements on Muscle Mass and Activities of Daily Living in Elderly Rehabilitation Patients with Decreased Muscle Mass: A Randomized Controlled Trial. J. Nutr. Health Aging.

[197] Almeida T.M.d., Gomes L.M.S., Afonso D., Magnoni D., Mota I.C.P., França J.Í.D., Silva R.G.d. (2020). Risk factors for oropharyngeal dysphagia in cardiovascular diseases. J. Appl. Oral Sci. Rev. FOB.

[198] Neloska L., Damevska K., Nikolchev A., Pavleska L., Petreska-Zovic B., Kostov M. (2016). The Association between Malnutrition and Pressure Ulcers in Elderly in Long-Term Care Facility. Open Access Maced. J. Med. Sci..

[199] Byeon H. (2019). Predicting the Swallow-Related Quality of Life of the Elderly Living in a Local Community Using Support Vector Machine. Int. J. Environ. Res. Public Health.

[200] Wakabayashi H., Kishima M., Itoda M., Fujishima I., Kunieda K., Ohno T., Ogawa S. (2021). Diagnosis and Treatment of Sarcopenic Dysphagia: A Scoping Review. Dysphagia.

[201] Di Pede C., Mantovani M.E., Del Felice A., Masiero S. (2016). Dysphagia in the elderly: Focus on rehabilitation strategies. Aging Clin. Exp. Res..

[202] Alghadir A.H., Zafar H., Al-Eisa E.S., Iqbal Z.A. (2017). Effect of posture on swallowing. Afr. Health Sci..

[203] Namiki C., Hara K., Tohara H., Kobayashi K., Chantaramanee A., Nakagawa K., Minakuchi S. (2019). Tongue-pressure resistance training improves tongue and suprahyoid muscle functions simultaneously. Clin. Interv. Aging.

[204] Cichero J.A.Y., Lam P., Steele C.M., Hanson B., Chen J., Dantas R.O., Stanschus S. (2017). Development of International Terminology and Definitions for Texture-Modified Foods and Thickened Fluids Used in Dysphagia Management: The IDDSI Framework. Dysphagia.

[205] Steele C.M., Namasivayam-MacDonald A.M., Guida B.T., Cichero J.A., Duivestein J., Hanson B., Riquelme L.F. (2018). Creation and Initial Validation of the International Dysphagia Diet Standardisation Initiative Functional Diet Scale. Arch. Phys. Med. Rehabil..

[206] Chen K.-C., Lee T.-M., Wu W.-T., Wang T.-G., Han D.-S., Chang K.-V. (2021). Assessment of Tongue Strength in Sarcopenia and Sarcopenic Dysphagia: A Systematic Review and Meta-Analysis. Front. Nutr..

[207] Vose A., Nonnenmacher J., Singer M.L., González-Fernández M. (2014). Dysphagia Management in Acute and Sub-acute Stroke. Curr. Phys. Med. Rehabil. Rep..

[208] Carnaby G., Crary M. (2007). Examining the Evidence on Neuromuscular Electrical Stimulation for Swallowing. Arch. Otolaryngol.—Head Neck Surg..

[209] Carnaby-Mann G., Crary M.A., Schmalfuss I., Amdur R. (2012). ‘’Pharyngocise’’: Randomized controlled trial of preventative exercises to maintain muscle structure and swallowing function during head-and-neck chemoradiotherapy. Int. J. Radiat. Oncol. Biol. Phys..

[210] Carnaby-Mann G.D., Crary M.A. (2010). McNeill dysphagia therapy program: A case-control study. Arch. Phys. Med. Rehabil..

[211] Crary M.A., Carnaby G.D., LaGorio L.A., Carvajal P.J. (2012). Functional and physiological outcomes from an exercise-based dysphagia therapy: A pilot investigation of the McNeill Dysphagia Therapy Program. Arch. Phys. Med. Rehabil..

[212] National Institute on Aging, National Institutes of Health, U.S. Department of Health and Human Services (2008). Why Population. https://2001-2009.state.gov/documents/organization/81775.pdf.

[213] Marcenes W., Kassebaum N.J., Bernabé E., Flaxman A., Naghavi M., Lopez A., Murray C.J. (2013). Global burden of oral conditions in 1990–2010: A systematic analysis. J. Dent. Res..

[214] Petersen P.E. (2009). Global policy for improvement of oral health in the 21st century--implications to oral health research of World Health Assembly 2007, World Health Organization. Community Dent. Oral Epidemiol..

[215] Preshaw P.M., Henne K., Taylor J.J., Valentine R.A., Conrads G. (2017). Age-related changes in immune function (immune senescence) in caries and periodontal diseases: A systematic review. J. Clin. Periodontol..

[216] López R., Smith P.C., Göstemeyer G., Schwendicke F. (2017). Ageing, dental caries and periodontal diseases. J. Clin. Periodontol..

[217] Azarpazhooh A., Leake J.L. (2006). Systematic review of the association between respiratory diseases and oral health. J. Periodontol..

[218] Martinez-Herrera M., Silvestre-Rangil J., Silvestre F.J. (2017). Association between obesity and periodontal disease. A systematic review of epidemiological studies and controlled clinical trials. Med. Oral Patol. Oral Cir. Bucal..

[219] Dizdar O., Hayran M., Guven D.C., Yılmaz T.B., Taheri S., Akman A.C., Berker E. (2017). Increased cancer risk in patients with periodontitis. Curr. Med. Res. Opin..

[220] Wu B., Fillenbaum G.G., Plassman B.L., Guo L. (2016). Association Between Oral Health and Cognitive Status: A Systematic Review. J. Am. Geriatr. Soc..

[221] Tonsekar P.P., Jiang S.S., Yue G. (2017). Periodontal disease, tooth loss and dementia: Is there a link? A systematic review. Gerodontology.

[222] Zarb G.A. (1983). The edentulous milieu. J. Prosthet. Dent..

[223] Niesten D., van Mourik K., van der Sanden W. (2013). The impact of frailty on oral care behavior of older people: A qualitative study. BMC Oral Health.

[224] Eberhard J., Jepsen S., Jervøe-Storm P.M., Needleman I., Worthington H.V. (2015). Full-mouth treatment modalities (within 24 hours) for chronic periodontitis in adults. Cochrane Database Syst. Rev..

[225] Manresa C., Sanz-Miralles E.C., Twigg J., Bravo M. (2018). Supportive periodontal therapy (SPT) for maintaining the dentition in adults treated for periodontitis. Cochrane Database Syst. Rev..

[226] Castro-Calderón A., Roccuzzo A., Ferrillo M., Gada S., González-Serrano J., Fonseca M., Molinero-Mourelle P. (2021). Hyaluronic acid injection to restore the lost interproximal papilla: A systematic review. Acta Odontol. Scand..

[227] Roccuzzo A., Molinero-Mourelle P., Ferrillo M., Cobo-Vázquez C., Sanchez-Labrador L., Ammendolia A., de Sire A. (2021). Type I Collagen-Based Devices to Treat Nerve Injuries after Oral Surgery Procedures. A Systematic Review. Appl. Sci..

[228] Togni L., Mascitti M., Santarelli A., Contaldo M., Romano A., Serpico R., Rubini C. (2019). Unusual Conditions Impairing Saliva Secretion: Developmental Anomalies of Salivary Glands. Front. Physiol..

[229] Davies A.N., Shorthose K. (2007). Parasympathomimetic drugs for the treatment of salivary gland dysfunction due to radiotherapy. Cochrane Database Syst. Rev..

[230] Fedele S., Wolff A., Strietzel F., López R.M., Porter S.R., Konttinen Y.T. (2008). Neuroelectrostimulation in treatment of hyposalivation and xerostomia in Sjögren's syndrome: A salivary pacemaker. J. Rheumatol..

[231] Sugiura Y., Soga Y., Yamabe K., Tsutani S., Tanimoto I., Maeda H., Takashiba S. (2010). Total bacterial counts on oral mucosa after using a commercial saliva substitute in patients undergoing hematopoietic cell transplantation. Support. Care Cancer.

[232] Assery M.K.A. (2019). Efficacy of Artificial Salivary Substitutes in Treatment of Xerostomia: A Systematic Review. J. Pharm. Bioallied Sci..

[233] Vadcharavivad S., Boonroung T. (2013). Original article. Effects of two carboxymethylcellulose-containing saliva substitutes on post-radiation xerostomia in head and neck cancer patients related to quality of life. Asian Biomed..

[234] Ameri A., Heydarirad G., Rezaeizadeh H., Choopani R., Ghobadi A., Gachkar L. (2016). Evaluation of Efficacy of an Herbal Compound on Dry Mouth in Patients with Head and Neck Cancers: A Randomized Clinical Trial. J. Evid. Based Complement. Altern. Med..

[235] Montaldo L., Montaldo P., Papa A., Caramico N., Toro G. (2010). Effects of saliva substitutes on oral status in patients with Type 2 diabetes. Diabet. Med..

[236] Abt E., Carr A.B., Worthington H.V. (2012). Interventions for replacing missing teeth: Partially absent dentition. Cochrane Database Syst. Rev..

[237] Marra R., Acocella A., Alessandra R., Ganz S.D., Blasi A. (2017). Rehabilitation of Full-Mouth Edentulism: Immediate Loading of Implants Inserted with Computer-Guided Flapless Surgery Versus Conventional Dentures: A 5-Year Multicenter Retrospective Analysis and OHIP Questionnaire. Implant. Dent..

[238] Allen P.F., McMillan A.S., Walshaw D. (2001). A patient-based assessment of implant-stabilized and conventional complete dentures. J. Prosthet. Dent..

[239] Ornstein K.A., DeCherrie L., Gluzman R., Scott E.S., Kansal J., Shah T., Soriano T.A. (2015). Significant unmet oral health needs of homebound elderly adults. J. Am. Geriatr. Soc..

[240] Chen X., Kistler C.E. (2015). Oral Health Care for Older Adults with Serious Illness: When and How?. J. Am. Geriatr. Soc..

[241] Paquette D.W. (2003). The periodontal infection-systemic disease link: A review of the truth or myth. J. Int. Acad. Periodontol..

[242] Borgnakke W.S., Ylöstalo P.V., Taylor G.W., Genco R.J. (2013). Effect of periodontal disease on diabetes: Systematic review of epidemiologic observational evidence. J. Periodontol..

[243] Stoffel L.M.B., Muniz F.W.M.G., Colussi P.R.G., Rösing C.K., Colussi E.L. (2018). Nutritional assessment and associated factors in the elderly: Apopulation-based cross-sectional study. Nutrition.

[244] Kossioni A.E., Hajto-Bryk J., Maggi S., McKenna G., Petrovic M., Roller-Wirnsberger R.E., Müller F. (2018). An Expert Opinion from the European College of Gerodontology and the European Geriatric Medicine Society: European Policy Recommendations on Oral Health in Older Adults. J. Am. Geriatr. Soc..

[245] Cumpston M., Li T., Page M.J., Chandler J., Welch V.A., Higgins J.P., Thomas J. (2019). Updated guidance for trusted systematic reviews: A new edition of the Cochrane Handbook for Systematic Reviews of Interventions. Cochrane Database Syst. Rev..

